# Supplementary material for: SOCS1 Inhibits IL-6-Induced CD155 Overexpression in Lung Adenocarcinoma
Source: Int J Mol Sci. 2024 Nov 12;25(22):12141. doi: 10.3390/ijms252212141 (PMC11595078; doi:10.3390/ijms252212141)
Supplement: Supplementary file 1 [file ijms-25-12141-s001.zip › ijms-3265225-supplementary.pdf]

SOCS1 Inhibits IL-6-Induced CD155 Overexpression in Lung Adenocarcinoma

Mario Marroquin-Muciño [1,2,†], Jesus J. Benito-Lopez [1,3,†], Mario Perez-Medina [1,2], Dolores Aguilar-Cazares [1], Miriam Galicia-Velasco [1], Rodolfo Chavez-Dominguez [1], Sergio E. Meza-Toledo [2], Manuel Meneses-Flores [1,4], Angel Camarena [5] and Jose S. Lopez-Gonzalez [1,*]

1 Laboratorio de Cancer Pulmonar, Departamento de Enfermedades Cronico-Degenerativas, Instituto Nacional de Enfermedades Respiratorias "Ismael Cosio Villegas", Mexico City 14080, Mexico; mario.mm@ciencias.unam.mx (M.M.-M.); mperezm1518@alumno.ipn.mx (M.P.-M.); daguilarc@iner.gob.mx (D.A.-C.); miriam.galicia@iner.gob.mx (M.G.-V.); rodolfo_chvz@comunidad.unam.mx (R.C.-D.); manuelmeneses707@gmail.com (M.M.-F.)
2 Laboratorio de Quimioterapia Experimental, Departamento de Bioquimica, Escuela Nacional de Ciencias Biologicas, Instituto Politecnico Nacional, Mexico City 11340, Mexico; smezat@ipn.mx
3 Posgrado en Ciencias Biologicas, Universidad Nacional Autonoma de Mexico, Mexico City 04510, Mexico
4 Departamento de Patologia, Instituto Nacional de Enfermedades Respiratorias "Ismael Cosio Villegas", Mexico City 14080, Mexico
5 Laboratorio de Inmunobiologia y Genetica, Instituto Nacional de Enfermedades Respiratorias "Ismael Cosio Villegas", Mexico City 14080, Mexico; ang_edco@yahoo.com.mx
* Correspondence: sullivan.lopez@iner.gob.mx
† These authors contributed equally to this work.

Supplementary Materials

Supplementary Table S1. DsiRNA sequences for SOCS1.

Name	Sequence	Sense
hs.Ri.SOCS1.13.1	5' rArGrUrCrArGrUrUrUrArGrGrUrArArUrArArArCrUrUrUAT 3'	+
	5' rArUrArArArGrUrUrUrArUrUrArCrCrUrArArArCrUrGrArCrUrUrU 3'	-
hs.Ri.SOCS1.13.2	5' rGrGrUrArArUrArArArCrUrUrUrArUrUrArUrGrArArArGTT 3'	+
	5' rArArCrUrUrUrCrArUrArArUrArArArGrUrUrUrArUrUrArCrCrUrA 3'	-
hs.Ri.SOCS1.13.3	5' ArGrUrCrArGrUrUrUrArGrGrUrArArUrArArArCrUrUrArT 3'	+
	5' rArUrArArArGrUrUrUrArUrUrArCrCrUrArArArCrUrGrArCrUrUrU 3'	-

Supplementary Table S2. Demographic and clinical characteristics of the lung adenocarcinoma cohort employed for tissue analysis.

**Total group**		**n = 39**
Age (years)		
	median	61.5
	range	24–87
Female		24
Male		15
Smoking habits		
	Yes	12
	No	27
Clinical Stage		
	I	2
	II	6
	III	7
	IV	24
Histologic subtypes		
	Acinar	14
	Papillary	4
	Lepidic	9
	Solid	8
	Micropapillary	4
Activating EGFR mutation		
	Yes	13
	No	26

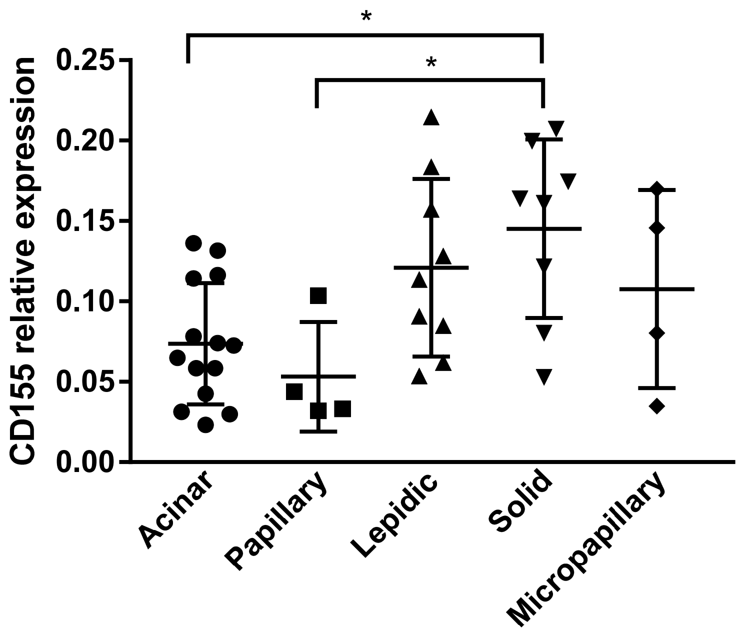

Supplementary Figure S1. Relative CD155 expression among LUAD histologic subtypes. CD155 expression in the predominantly solid subtype is higher, compared to predominantly acinar and papillary subtypes. Data are shown as median ± SD. * $p < 0.05$.
